# Dr. John Herr Musser

**Published:** 1912-05

**Authors:** 


Dr. John Herr Musser, whose father, grandfather, and great grandfather were also physicians, was born June 22, 1856, at Strasbourg, Pa. He graduated in medicine at the University of Pennsylvania in 1877 and practiced in Philadelphia up to the time of his death, which occurred April 3, 1912, of angina pectoris. From quiz master, he was promoted, step by step, to the professorship of clinical medicine at the University of Pennsylvania, in spite of the old precedent of selection from prominent Philadelphia families, a precedent from which the University has departed more and more often of late years. Aside from his college and hospital connections, Dr. Musser was President of the A. M. A. 1903-4, and of many other organizations, at one time or another. His Medical Diagnosis, is a standard and his services as editor in Nothnagel's cyclopaedia and as joint author with A. O. J. Kelly in preparing a system of therapeutics, as well as his contributions to current literature, will live after him. He was also active in various phases of philanthropy and good citizenship. One of his legacies to the profession is his brain, which is the property of the Wistar Institute of Anatomy but which will be subjected to the critical study of a number of institutions. In passing, it may be said that the same institution also possesses the brain of the late Dr. Waldemar Koch of Chicago. What makes this legacy particularly valuable, in Dr. Musser's case, is the absolute normality of his mind. He had none of the eccentricities of genius, no one would have called him brilliant, he was evenly balanced in his interests, not only in the sense that he was not a specialist except as the word internist indicates a lopping off of certain forms of practice, but in the sense that as a physician, he was both practical and scientific. He wrote and read much but he was neither book-worm nor author in the exclusive sense. He was a teacher, but a teacher of things that he had seen. More than this, his symmetric medical development was, itself, balanced by his development as a moral, sensible, kindly man, of broad sympathies and interests, yet with too stable judgment to allow us to designate him as versatile. With an acquaintance beginning in Philadelphia as a student, many years ago, and kept up partly by correspondence, partly by meetings of societies, we feel a keen sense of personal loss in Dr. Musser's death. His greatness grows with contemplation and nearness of approach, like that of a massive pillared temple which, at first glance, seems of ordinary size, because no one part is exaggerated and there is no succession of stories to afford a measure.

